# Drug-Induced Podocytopathies: Report of Four Cases and Review of the Literature

**DOI:** 10.3390/life13061264

**Published:** 2023-05-26

**Authors:** Diamanto Athanasopoulou, Sophia Lionaki, Chrysanthi Skalioti, George Liapis, Panayiotis Vlachoyiannopoulos, Ioannis Boletis

**Affiliations:** 1 Department of Nephrology and Transplantation, National and Kapodistrian University of Athens, Laiko Hospital, 115 27 Athens, Greece; c_skalioti@yahoo.com (C.S.); inboletis@gmail.com (I.B.); 2 Department of Nephrology, National and Kapodistrian University of Athens, Attiko Hospital, 124 62 Athens, Greece; sofia.lionaki@gmail.com; 3 Department of Pathology, National and Kapodistrian University of Athens, Laiko Hospital, 115 27 Athens, Greece; gliapis@gmail.com; 4 Department of Pathophysiology, National and Kapodistrian University of Athens, Laiko Hospital, 115 27 Athens, Greece; pvlah@med.uoa.gr

**Keywords:** drug-induced glomerulonephritis, drug-induced podocytopathies, tamoxifen, penicillamine, pembrolizumab, axitinib

## Abstract

Kidney injury due to medications is a well-known clinical entity. Although drug-induced tubulointerstitial disease is commonly encountered, there are few reports in the literature associated with glomerular injury due to medications. The recognition of this type of kidney injury is crucial, as rapid discontinuation of the offending agent is critical to maximizing the likelihood of quick and effective renal function recovery. In this article, we present four cases that presented with nephrotic syndrome and were diagnosed with biopsy-proven podocytopathies, associated with exposure to a certain medication. All of them experienced complete resolution of nephrotic syndrome within days or weeks after discontinuation of the offending drug. We also present the data, which were found in a Medline search from the year 1963 until the present, regarding cases with podocytopathies associated with penicillamine, tamoxifen and the combination of pembrolizumab-axitinib, including only adult cases from the English literature. The Medline search revealed nineteen cases of penicillamine-induced minimal-change disease (MCD), one case of tamoxifen-induced MCD, and none associated with pembrolizumab-axitinib therapy. We also searched for the largest studies and meta-analyses regarding drug-induced podocytopathies after a Medline search from 1967 to the present of the English literature.

## 1. Introduction

Increasing evidence supports the hypothesis that podocyte injury can result from a single causative agent or a combination of multiple factors acting with complex mechanisms, making multifactoriality relevant in the pathophysiology of these disorders. Medications cause approximately 20% of community- and hospital-acquired episodes of acute kidney injury (AKI), and among older adults, the incidence of drug-induced nephrotoxicity is as high as 66% [1,2]. Medications have the capacity to damage cells within all compartments of the kidney. Tubulointerstitial injury is most commonly mentioned, whereas less attention has been paid to glomerular injury. However, recent advances in the study of glomerular physiology and metabolism have increased our understanding of the pathogenesis of drug-induced glomerular disease. In the glomerulus, both the cells and the subepithelial space can be affected. The podocytes, the endothelial cells and the mesangial cells can be affected. There are four major mechanisms of glomerular toxicity: cytotoxic or charge-related injury to the filtration barrier, toxicity related to accumulation of a xenobiotic within the glomerular compartment based on pharmacologic or off-target activity, glomerulosclerosis and immune-mediated injury [3]. Drug-induced podocytopathies typically include minimal-change disease (MCD) and focal segmental glomerulosclerosis (FSGS), which are clinically manifested with proteinuria and nephrotic syndrome with or without AKI. Known representatives from the literature are pamidronate, IFN, TKIs, lithium, NSAIDs, quinolones, rifampicin, ipilimumab, nivolumab and pembrolizumab for MCD and tyrosine kinase inhibitors (TKIs), sirolimus, pamidronate, anabolic steroids, lithium, IFN and nivolumab for FSGS [2]. Endothelial cell injury appears as thrombotic microangiopathy, clinically characterized by microangiopathic hemolytic anemia. Therapeutic agents affecting endothelial cells include gemcitabine, mitomycin C, calcineurin inhibitors, sirolimus, everolimus, IFN, vincristine, opioids, proteasome inhibitors, valproic acid, IVIg, quinine, IFN, thienopyridines, oxaliplatin, quetiapine, gemcitabine, penicillin and sulfisoxazole [2]. Mesangial cell injury emerges as glomerulosclerosis and IgA nephropathy, with common examples being vancomycin, carbamazepine, ceftriaxone, metronidazole, cyclosporine, acetaminophen, amiodarone, furosemide, nivolumab, ipilimumab and pembrolizumab [2]. Subepithelial-space injury, caused by medications such as gold, penicillamine, captopril, NSAIDs, gefitinib, nivolumab, adalimumab, celecoxib and lithium, is manifested as membranous nephropathy (MN) [2].

## 2. Case 1 Description: MCD Associated with Tamoxifen

A 49-year-old female presented in 2017 with 1-week history of peripheral edema, increase in body weight up to 10 kg and intermittent unilateral lumbar pain. Her past medical history was significant for hepatitis B infection; rheumatoid arthritis diagnosed 12 years prior to presentation, treated with methotrexate and steroids for four years and adalimumab for two more years; and in situ breast carcinoma 6 years prior to presentation, for which she had surgical excision and radiotherapy and was on tamoxifen afterwards. She was not on any NSAIDs. Physical examination revealed bilateral pitting leg edema up to the thighs and reduced air entry bibasally. Her blood pressure was 117/70 with 64 pulses, without any orthostatic signs. Laboratorial findings were remarkable for nephrotic-range proteinuria (9.7 g/24 h) without microscopic hematuria, eosinophiluria or aseptic pyuria; low serum albumin (1.6 g/dL); and hyperlipidemia (total cholesterol 459 mg/dL, triglycerides 282 mg/dL, low high-density lipoprotein 322 mg/dL). Hemoglobin, white blood cells including eosinophils, C-reactive protein (CRP) and renal and hepatic function were normal. Serum protein electrophoresis was normal, and no monoclonal component was detected on immunofixation. Serum-free light chains, complement compounds (C3 and C4), and serological tests all were normal. Blood and urine cultures were negative. Hepatitis B surface antigen and anti-HBc were positive, and anti-hepatitis C virus and anti-HIV antibodies were negative. Thyroid hormones and CA15-3 were within normal limits. Echocardiogram and renal ultrasound were negative.

**Figure 1:**
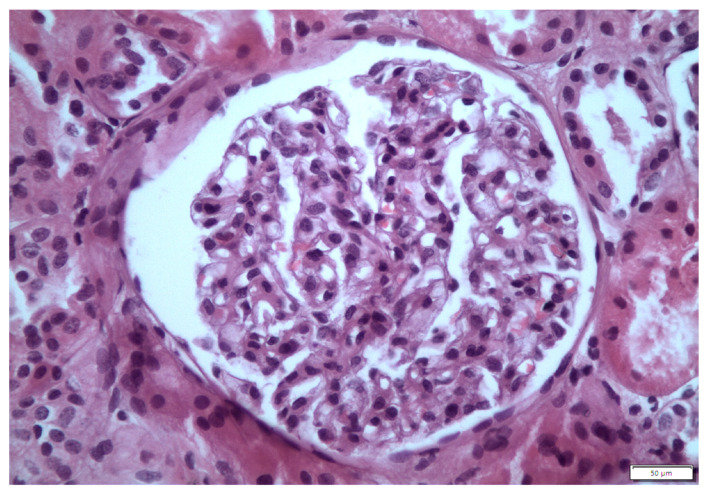
Light microscopy: glomeruli were unremarkable.

**Figure 2:**
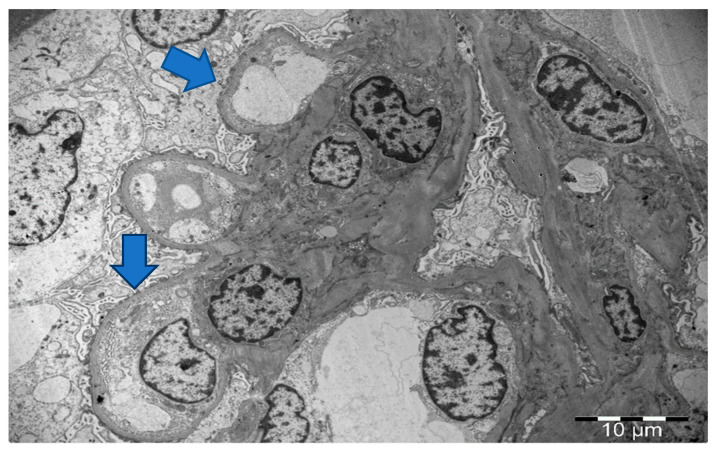
Electron microscopy: podocyte activation and foot processes effacement (blue arrows) with microvilli transformation, without substantial immune-complex deposits in glomerular basement membranes or mesangium.

A kidney biopsy was performed, and fourteen glomeruli were obtained. One had glomerulosclerotic appearance, with the rest having normal size without hypercellularity, slightly thickened capillary walls and normal mesangium (Figure 1). Glomerular basement membrane (GBM) was normal. Tubular atrophy and interstitial fibrosis were presented in 20% of renal parenchyma. Slight arteriosclerosis was documented. Congo red staining was negative. Immunofluorescence was negative. Electron microscopy revealed loss of podocyte foot processes with microvilli (Figure 2).

To exclude secondary causes associated with MCD, a total-body computed tomography was performed with no findings.Thyroid ultrasound showed only increased generalized vasculature, and breast magnetic tomography, gastroscopy and colonoscopy were normal. The patient was also evaluated by an endocrinologist and was started on thyroxine 50 mcg, while oncology consultation was negative for breast cancer exacerbation or any new cancer development. Exclusion of all other causes led to tamoxifen discontinuation with gradual but significant improvement of proteinuria, ending in complete resolution of nephrotic syndrome within 3 weeks of cessation. 

## 3. Case 2 Description: MCD Associated with D-Penicillamine

A 46-year-old female presented with nephrotic syndrome associated with new-onset significant peripheral edema and 1-day history of left-sided pleuritic chest pain. Her past medical history was significant for latent tuberculosis (positive Mantoux test), hypothyroidism for almost 10 years and a 6-month history of scleroderma associated with Raynaud’s phenomenon, fingertip ulcers, generalized weakness and skin telangiectasis. Medications at presentation included isoniazid, thyroxine, methylprednisolone at 8 mg per day, recently started (5 months prior to presentation) D-penicillamine (200 mg twice per day) and bosentan (dual endothelin receptor antagonist) for the last 3 days prior to presentation. She was not on any NSAIDs.

**Figure 3:**
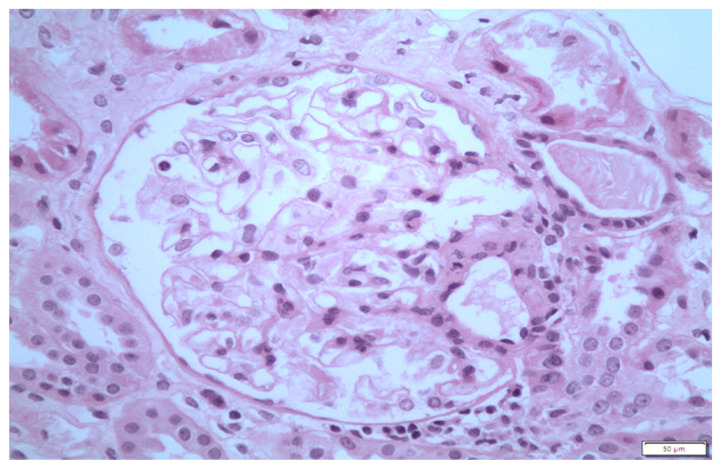
Light microscopy: glomeruli showed no essential changes.

**Figure 4:**
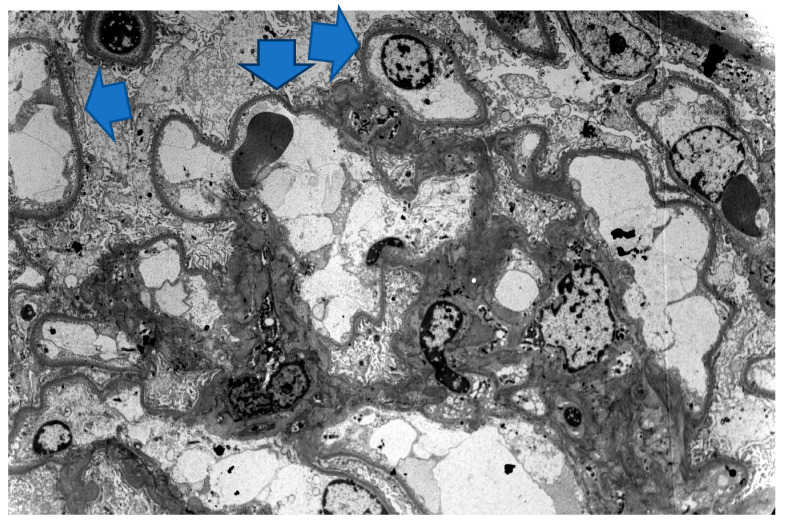
Electron microscopy: diffuse foot processes effacement (blue arrows) with microvilli transformation; no immune-complex deposits were found. Magnification: 2200×.

On physical examination, she exhibitedbilateral pitting leg edema, reduced air entry on the left base and scleroderma-like changes, with ulcers on both hands. Laboratory findings showed nephrotic-range proteinuria (24 g/24 h) with microscopic hematuria (RBCs 4-5 per hpf), low serum albumin (2 g/dL) and hyperlipidemia (total cholesterol 338 mg/dL, triglycerides 156 mg/dL, low high-density lipoprotein 187 mg/dL). Urinalysis was negative for white cells and white-cell casts. Hematological profile, CRP and renal, hepatic and thyroid function were normal. Human immunodeficiency virus (HIV) and hepatitis screen were negative. A kidney biopsy was scheduled and D-penicillamine was withheld, as it was associated with new-onset nephrotic syndrome. Furthermore, methylprednisolone dose was increased to 32 mg/day. Discontinuation of D-penicillamine in combination with glucocorticoids therapy resulted in significant decrease in proteinuria, which ended up in normal range within the next 15 days. Despite decrease in proteinuria, the kidney biopsy was carried out, showing 55 glomeruli with normal size and mesangium without hypercellularity (Figure 3). GBM was normal. Tubular atrophy and interstitial fibrosis were present in 15% of renal parenchyma. Slight arteriosclerosis of the large vessels was documented. Immunofluorescence was negative. A diagnosis of a recently depressed MCD was made, as confirmed by electron microscopy (Figure 4).

## 4. Case 3 Description: D-Penicillamine-Associated MCD

A 75-year-old female was referred to our clinic by her treating rheumatologist in 2012, with 15-day history of lower limb and bilateral eyelid edema. Her past medical history included osteopenia, osteoarthritis and 1-year history of scleroderma and Raynaud’s phenomenon, for which she was given D-penicillamine and bosentan. Other medications included risedronate, atorvastatin, enalapril, rabeprazole and low molecular heparin. She was not on any NSAIDs. On physical examination, she had bilateral pitting leg edema and eyelid edema. Laboratory findings showed nephrotic-range proteinuria (6.4 g/24 h) without microscopic hematuria, low serum albumin (2 g/dL) and hyperlipidemia (total cholesterol 400 mg/dL, triglycerides 265 mg/dL, low high-density lipoprotein 280 mg/dL). Urine sediment was negative for eosinophils and white-cell casts. Hematological profile including eosinophils, CRP and renal, hepatic and thyroid function, were normal. Immunologic profile was normal except for antinuclear antibodies (ANA >1:640). Screening for HIV and hepatitis was negative. Cessation of D-penicillamine was followed by gradual but significant improvement of nephrotic syndrome until complete resolution, i.e., proteinuria reduced to normal range within the next 10 days, which remains normal up to the present. The kidney biopsy revealed 26 glomeruli of normal size without hypercellularity, with only 2 of them having chronic lesions (sclerosis).The mesangium and GBMwere normal. Tubular atrophy and interstitial fibrosis were present in 15% of renal parenchyma. Arterioles were documented with mild partial segmental hyalinosis and thickening of inner wall. There were not any onion-skin-like changes scleroderma related in vasculature. Immunofluorescence was negative. The diagnosis was MCD, as widespread foot processes effacement was found by electron microscopy.

## 5. Case 4 Description: Pembrolizumab-Axitinib-Induced MCD

A 74-year-old man presented in 2020 with nephrotic syndrome and acute kidney injury (serum creatinine 5.8 mg/dL from 1.8 mg/dL baseline creatinine), which was found during workup for generalized weakness. His past medical history included renal cell carcinoma (RCC) with left nephrectomy (7 months before admission) on combined therapy with axitinib (tyrosine kinase inhibitor–anti-VEGF) and an ICPI pembrolizumab administered intravenously every 3 weeks. He also had asymptomatic rheumatoid arthritis (not on treatment) and hypertension on amlodipine.

**Figure 5:**
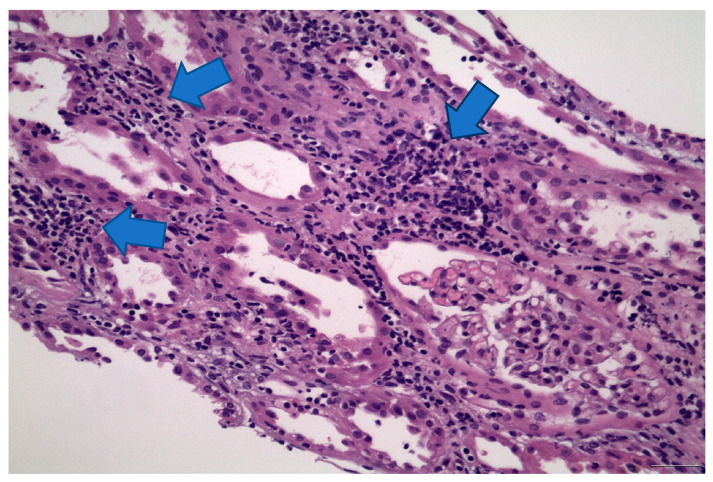
Light microscopy: acute tubular injury, in association with tubulointerstitial nephritis, as manifested by the aggregations of lymphocytes and monocytes into the interstitium (blue arrows), while glomeruli showed no abnormalities. Magnification: H&E 200×.

Physical examination was significant for revealed bilateral pitting leg edema. Laboratory findings were remarkable for nephrotic-range proteinuria (18 g/24 h) without microscopic hematuria or eosinophiluria, low serum albumin (1.9 g/dL), hyperlipidemia (total cholesterol 467 mg/dL, triglycerides 342 mg/dL) and raised creatinine (5.8 mg/dL). Serum protein electrophoresis showed elevated a2 proteins, but all the rest of the immunological tests were normal. Hemoglobin, white blood cells including eosinophils, CRP and hepatic function were normal. Hepatitis screen showed previous HBV infection. Echocardiogram was normal. The kidney biopsy specimen included only four glomeruli, which had normal size, without any glomerulosclerotic appearance or hypercellularity. Mesangium appeared with mild hypercellularity. Tubular interstitial necrosis in connection with acute interstitial nephritis was present, as diagnosed from tubular dilatation, flattened tubular epithelial cells and edema, and lymphocyte infiltration and tubulitis. GMB was normal. Slight arteriosclerosis was documented. Congo red staining was negative. Immunofluorescence showed IgM+2, IgA trace, C3+1 and L light chain +1. The histological findings were suggestive of tubular interstitial necrosis, acute interstitial nephritis and MCD (Figure 5). The sample was inadequate for electron microscopy.

Chemotherapy and immunotherapy were discontinued, and oral glucocorticoids (prednisolone 1 mg/kg) were started, with significant improvement of kidney function and proteinuria and complete remission after 3 months. However, three months after his hospital discharge, while on complete remission, he was restarted on another type of VEGF inhibitor (pazopanib) by the treating oncologist, leading to relapse of nephrotic syndrome nearly 1 month post treatment initiation. A new course of glucocorticoids was given, and pazopanib was discontinued. Two months later, immunosuppression was switched to cyclosporine and glucocorticoids were discontinued, due to adverse reactions. A partial remission was achieved 3 months post pazopanib discontinuation.

## 6. Discussion

Irrespective of the primary insult, podocyte damage, detachment and loss increase the permeability of the glomerular filtration barrier, causing proteins to be filtered intourine. According to this, the clinical hallmark of podocytopathies is proteinuria, ranging from subnephrotic to nephrotic range. In this article, we describe twocases of drug-induced nephrotic syndrome associated with initiation of D-penicillamine with histological confirmation of MCD. The duration of treatment of the first patient before the nephrotic syndrome diagnosis was 6 months, whereas the duration of the second patient was 12 months. Both had normal renal function without a history of proteinuria and achieved complete remission of nephrotic syndrome within a few weeks post D-penicillamine discontinuation, without any specific therapy. Penicillamineis a medication primarily used for the treatment of Wilson’s disease [4,5] but also for cystinuria, rheumatoid arthritis, various heavy metal poisonings and scleroderma [6,7]. There are many side effects of penicillamine, with rash, loss of appetite, nausea, diarrhea and leucopenia being the most common, whereas nephropathy, hepatotoxicity, aplastic anemia [8], antibody-mediated myasthenia gravis [9], Lambert–Eaton myasthenic syndrome and drug-induced systemic lupus erythematosus [10] are some other possible adverse reactions. Proteinuria and nephrotic syndrome are the most commonly encountered features of renal pathology [11,12,13]. Proteinuria is the most common kidney-related adverse reaction due to penicillamine, with MN being the most common histological pattern, followed by MCD [11,12,13]. No appreciable changes to renal function have been noted. Penicillamine-associated nephropathy encompasses several other types of renal damage, due to different immunopathogenic mechanisms, such as mesangioproliferative glomerulonephritis, IgM nephropathy and focal proliferative glomerulonephritis [13,14,15]. Furthermore, rapidly progressive glomerulonephritis due to p-ANCA vasculitis has been associated with penicillamine treatment [16,17,18,19,20]. Known risk factors for the development of proteinuria include HLA-B8 and HLA-DR3 positive patients, prior gold-induced proteinuria, higher age and low titer of rheumatoid factor [13]. Pathogenesis is generally unclear. Penicillamine could act as a hapten, causing antibody formation and immune-complex deposition. It could also possibly alter polymeric proteins as a rheumatoid factor, enhancing the nephritogenic potential of existing rheumatoid immune complexes [11]. The duration of penicillamine treatment before the proteinuria diagnosis could be 3–12 months, whereas the duration before the diagnosis of nephrotic syndrome could be 2 weeks to 11 years [21]. The progression from proteinuria to nephrotic syndrome could happen in a few weeks or years [19]. The majority of patients were females at the age of 44, while the mean dose of penicillamine at diagnosis was 0.125 to 2 g. Out of 19 patients mentioned in the English literature with MCD and nephrotic syndrome associated with penicillamine, 2 received steroid treatment and achieved complete remission within 1–5 weeks, whereas the patients who had simply discontinued the drug had proteinuria reduction (<1 g/24 h) within 0.5 to 21 months [11,13]. In three studies, it was found that proteinuria increased even after drug discontinuation, with later reduction in some and no proper follow-up in others [11,22]. Two studies showed that 70% of patients with persistent proteinuria will end up with nephrotic syndrome [19,23]. There is no agreement on the level of proteinuria at which the drug should be stopped [19]. The conclusion regarding nephrotic syndrome treatment is that the use of glucocorticoids is generally unnecessary unless there is a need for faster improvement [13].

The next case described in this article refers to a drug-induced nephrotic syndrome associated with initiation of tamoxifen with histological confirmation of MCD. Tamoxifenis a selective estrogen receptor modulator used to prevent breast cancer in women and treat breast cancer in both women and men [24,25]. It acts as a selective estrogen receptor modulator, or as a partial agonist of the estrogen receptors. It binds to estrogen receptors competitively in tumor cells and other tissue targets, producing a nuclear complex that decreases DNA synthesis and inhibits estrogen effects. It has different activity in different tissues, so it has predominantly antiestrogenic effects in the breasts but predominantly estrogenic effects in the uterus and liver [26]. Several other medical uses are known, such as ovulation induction for the treatment of female infertility [27], treatment of male infertility, treatment of female dysmenorrhea, the prevention and treatment of gynecomastia [28] and the treatment of peripheral precocious puberty in both girls and boys [29]. Endometrial cancer [30], thromboembolism, hypertriglyceridemia and hepatotoxicity are the side effects written about in the literature. There are no clinical reports on the effects of tamoxifen on kidney disease. A study on mice suggested that tamoxifen may protect against diabetic glomerulosclerosis by modulating the phenotype of podocytes in diabetes. The study showed reduction in proteinuria in type II diabetic mice [31]. Another study revealed the use of tamoxifen in order to induce nephrin deletion in mice, with nephrin being a protein whose expression in a mature glomerulus can result in a slowly progressive disease that histologically resembles FSGS, a disease linked closely with podocyte depletion [32]. Tamoxifen was also used in another study, which elucidated the role of estrogen in the pathogenesis of childhood nephrotic syndrome. In that study, the cell proliferation of mouse renal podocytes and human primary renal podocytes was promoted by 17β-estradiol (E2), and apoptosis was inhibited by E2 and promoted by the E2 antagonist, tamoxifen [33]. Only one case report regarding nephrotic syndrome due to podocytopathy has been published [34].

The fourth case is related to nephrotic syndrome due to MCD 15 days post initiation of a combination therapy of vascular endothelial growth factor receptor inhibitor (anti-VEGF) and immune checkpoint inhibitor (ICPI) for a patient suffering from metastatic renal cell carcinoma. Our patient had complete remission 3 months post therapy discontinuation in combination with steroid treatment.

RCC is the 6th most common cancer type in men and the 10th most common in women. [35]. Most localized RCC cases can be surgically excised, but the development of distant metastases (20–40%) necessitates systemic treatment in the first 5 years after primary tumor surgery. Numerous tyrosine kinase inhibitors (TKIs), mammalian-target-of-rapamycin inhibitors (mTORis) and ICPIs have been recently developed. In recent years, combination therapies have also been approved, such as combinations of ICPIs and combinations of ICPIs with TKIs, due to their superior clinical outcomes over monotherapies [36,37]. Despite the fact that these new treatments have significantly improved clinical effect, they are not free of adverse reactions, including renal toxicities [38]. Axitinib, sunitinib, pazopanib, sorafenib and cabozantinib are TKIsused in RCC treatment. The above drugs interfere with the activity of more than one family of receptor tyrosine kinases, including VEGFR, platelet-derived growth factor receptor (PDGFR), fibroblast growth factor receptor (FGFR), and epidermal growth factor receptor (EGFR) [38]. The most frequent adverse reactions are gastrointestinal disturbance, skin toxicity, fatigue, hypertension and renal toxicity, clinically manifested from an asymptomatic proteinuria to renal failure [39]. CKD prior to the start of treatment does not necessarily increase the risk of kidney function deterioration [38]. Only a few patients found in the literature have been biopsied. The most common histopathological feature is thrombotic microangiopathy, followed by crescentic glomerulonephritis, FSGS, immune-complex-mediated glomerulonephritis, MCD and acute tubulointerstitial nephritis [39]. The presumable mechanisms of proteinuria are loss of endothelial fenestrations in the glomeruli, endothelial-cell cytoplasm swelling, podocyte damage, decreased expression of nephrin, narrowing or occlusion of capillary lumina by basement membrane and tubulointerstitial damage [39]. AKI, nephrotic-range proteinuria and thrombotic microangiopathy are generally considered reasons to discontinue therapy with TKIs, and reintroduction might be possible at lower doses [40]. Axitinib, which is the most selective drug, interacts with VEGFR-1, VEGFR-2 and VEGFR-3 [41]. A recent systematic review of anti-VEGF nephrotoxicity in RCC patients [39], including 27 clinical trials, 4 meta-analyses and 17 retrospective studies, revealed that in phase III/IV clinical trials, high-grade proteinuria (nephrotic-range proteinuria or nephrotic syndrome) associated with axitinib appeared only in 8.2% of patients in total, whereas all-grade proteinuria appeared in 11.64% of patients treated with first-line axitinib and in 20.74% of patients treated with second-line axitinib. In a Japanese study [42], 65.9% of patients without existing proteinuria developed proteinuria after a median therapy with axitinib as second line over 12 months. In the largest study with biopsy-confirmed podocytopathies, Izzedine et al. [43] present one patient on axitinib, five patients on sunitinib and two patients on sorafenib with metastatic RCC. None of them had concurrently thrombotic microangiopathy. The mean interval between therapy initiation and onset of proteinuria was 65 ± 50.5 days. All of them had been through unilateral nephrectomy but without any proteinuria before therapy initiation and appeared with nephrotic-range proteinuria (with or without nephrotic syndrome), and two of them also had AKI. All of them discontinued therapy and had angiotensin-converting-enzyme inhibitors (ACEi) or angiotensin II receptor blockers (ARB). Only one, unfortunately, received follow-up after three years, as everyone else died shortly due to cancer progression. The aforementioned case is the only axitinib-related podocytopathy found in the literature so far.

Immune checkpoint inhibitors have beenapproved for many malignancies, including melanoma, lung cancer, RCC andurothelial carcinoma. They are humanized antibodies that inhibit down-regulatory receptors on T-cells (i.e., cytotoxic T-lymphocyte antigen 4 and programmed cell death 1 [PD-1] and its ligand, PD-L1), allowing them to maintain their antitumor activity [44]. However, the activation of immune response is complicated by immune-related adverse events affecting any organ, with the most frequent being dermatitis, rash, vitiligo, colitis, pneumonitis, hypophysitis, hypothyroidism and other endocrinopathies [45]. Immune-related nephrotoxicity (tubulointerstitial—more common—and glomerular disease) is less common and found mainly in ICPI combination therapies [46]. The delayed onset supports the immunity relation rather than direct toxicity. The mechanism mediating glomerulopathy is still unclear [46,47]. Hypotheses include the development of autoantibodies against self-antigens present on tubular epithelial cells, mesangial cells or podocytes; the migration and activation of effector T-cells in renal tissue and the infiltration of other immune cells together with pro-inflammatory cytokine release; and the development, the proliferation and the aberrant activation of a clone of self-reactive T-cells [46]. A recent systematic review and meta-analysis [47], including 27 articles with 45 biopsy-confirmed cases of ICPI-associated glomerular disease from an inception cohort, revealed pauci-immune glomerulonephritis as the most common histopathological picture (27%), followed by podocytopathies (24%) and complement 3 glomerulonephritis (11%). IgA nephropathy, lupus-like nephritis, thrombotic microangiopathy and anti-glomerular basement membrane disease (anti-GBM) followed with much smaller percentages. Concomitant acute interstitial nephritis (AIN) was reported in 41% of patients. They were mainly males with a median age of 63 and various cancer types. They had basically normal baseline kidney function. The median time between the treatment initiation and diagnosis of glomerulopathy was 3 months, clinically appearing as mild or severe AKI and/or nephrotic-range proteinuria. Glomerular disease occurred as late as 7 months into treatment and even months post discontinuation. Most patients had ICPIs discontinued (88%), and nearly all received corticosteroid treatment (98%). However, the majority of patients had full (31%) or partial (42%) AKI recovery, but 19% remained dialysis-dependent. The incidence of ESKD was greater (19%) when compared with published data from ICPI-induced tubulointerstitial cases (9%). More specifically, in terms of podocytopathies, 11 cases were mentioned in total, with relatively preserved renal function but severe nephrotic-range proteinuria, with median 10.3 g/day. Out of them, 5 patients on pembrolizumab and 2 patients on ipilimumab were diagnosed with MCD, whereas 2 on nivolumab were diagnosed with FSGS. All of them were treated with prednisone at 1 to 2 mg/kg/day, except for 1 patient who received pulse steroids. Only 8 out of 11 achieved complete or partial proteinuria remission. One patient ended up with ESKD. Regarding restarting the same therapy, there is only an individualized approach and not any specific recommendation so far due to the small numberof cases.

Regarding the combination therapies, in clinical trials, the incidence of ICPI acute kidney injury was estimated to be higher in patients receiving combination ipilimumab/nivolumab therapy compared to monotherapies (pembrolizumab, nivolumab, ipilimumab) [48]. In a follow-up of a phase III trial, where pembrolizumab- axitinib combination was compared with sunitinib monotherapy in patients with advanced renal cell carcinoma, AKI was present in 5 of 429 patients (1.2%) and interstitial nephritis in 8 (1.9%) patients in the combination group, almost similar to the monotherapy group. In contrast, proteinuria was noted in 81 (19%) patients in the combination group, contrary to 51 patients (12%) in the monotherapy group [37]. Only two case reports regarding patients with RCC on pembrolizumab-axitinib combination therapy and renal toxicity have been published [49,50]. The first patient developed AKI and mild proteinuria after four pembrolizumab cycles. No biopsy was practiced, immunosuppression was held, 1 mg/kg prednisolone was initiated with gradual improvement, and only axitinib was restarted seven days later. The second case developed AKI and mild proteinuria 3 weeks post therapy initiation. Renal biopsy revealed acute interstitial nephritis and mild mesangial proliferative glomerulonephritis with diffuse mesangial deposit of IgM (3+). Therapy was held, 40 mg prednisolone was given, and axitinib was restarted later despite proteinuria persistence. There is not any case reported so far regarding nephrotic syndrome MCD related to this combination therapy. Our patient developed tubule-interstitial disease and glomerulopathy in 15 days post treatment, supporting that direct toxicity of axitinib is most likely related rather than pembrolizumab.

The largest studies and meta-analyses regarding biopsy-proven drug-induced podocytopathies based on a Medline search from 1967 to the present of the English literature are presented in Table 1.

The association between nonsteroidal anti-inflammatory drugs (NSAIDs) and glomerulopathies has long been recognized. Literature has shown that MCD and MN have been the most common findings [62,63,64]. They have been described both in combination with AIN [65] and as separate entities [63,64]. These glomerular diseases are not necessarily separate entities distinctly demarcated from each other and with separate mechanisms but rather exist on a continuous spectrum of renal responses to a state of hypersensitivity against medication [64]. In a recent case report [65], MCD, MN and AIN were found simultaneously in the same biopsy of a patient presenting with nephrotic syndrome nine months post NSAIDs use. However, primary MN could not be easily ruled out. Cases of nephrotic syndrome associated with NSAIDs’ use with AIN are neither drug-class-specific nor dose-dependent [62]. Ranvskov et al., in their study [63], demonstrated that the duration of therapy is strongly associated with the diagnosis, while relapses of proteinuria have been reported after re-exposure to NSAIDs [66].

The largest systematic observational matched patient-control study regarding the association between NSAIDs and nephrotic syndrome was conducted in 2019 by Bakhriansyah et al. [56]. They concluded that current use > 2 weeks, recent use (discontinuation 1–2 months before nephrotic syndrome diagnosis date) and past use (discontinuation > 2 months) were associated with a significantly higher risk of nephrotic syndrome compared with nonuse and that the risk disappeared after 2 years of discontinuation. The use of selective cyclooxygenase-2 (COX-2) inhibitors was not associated with a higher risk of nephrotic syndrome, although there was not a statistically significant trend among patients with a past use, in contrast to conventional NSAIDs. Unfortunately, a kidney biopsy had been performed in only 11% of included patients. Findings, however, were similar when only cases with information on kidney biopsy were used and after adjusting for confounding factors. Among the glomerulopathies, MN and FSGS were the most common findings, with MCD following in fifth place after diffuse crescentic and diffuse mesangiocapillary glomerulonephritis.

In their meta-analysis, Perazella et al. presented 22 patients with podocytopathies in association with pamidronate and zoledronate therapy [53]. All 21 patients had received pamidronate intravenously for 2 months up to 2 years until the clinical picture appearance (17 had nephrotic syndrome, 3 had nephrotic-range proteinuria, 1 had non-nephrotic proteinuria, and 15 patients among those with nephrotic syndrome and nephrotic-range proteinuria had renal insufficiency). Renal biopsy findings revealed that 3 patients had MCD, 4 had FSGS and 14 had collapsing FSGS. Out of the 20 patients that had pamidronate withdrawal, 4 had complete remission, 5 showed a decline in proteinuria and 2 had persistent nephrotic syndrome. Out of them, seven showed creatinine reduction, whereas two showed a rise in creatinine and five ended up on dialysis. Regarding the MCD patients, in more details, two had complete remission, whereas the third ended up on dialysis after stopping the drug. Only a single case of nephrotic syndrome and renal insufficiency related to collapsing FSGS following treatment with zoledronate has been found in the literature. This patient ended up on dialysis post drug withdrawal. It was revealed that nephrotoxicity associated with intravenous bisphosphonates is a phenomenon that is dose- and infusion-time-dependent. Experiments have shown that the therapeutic effect of bisphosphonates on osteoclasts may have an identical mechanism to the toxic effect on the kidney, due todisruption of podocyte cytoskeleton assembly within osteoclasts and inhibition of farnesyl diphosphate in proximal tubular cells [67].

Herlitz et al., in 2010 [55], studied 10 bodybuilders who had long-term anabolicsteroid abuse(>8 years). Three patients were presented with nephrotic syndrome, six had nephrotic-range proteinuria, and one had non-nephrotic-range proteinuria. All of them had renal insufficiency. Renal biopsy revealed FSGS in nine patients, four of whom also had glomerulomegaly, and glomerulomegaly alone in one patient. Among 8 patients with mean follow-up of 2.2 years, one progressed to ESKD shortly after diagnosis, the other 7 received a renin-angiotensin system blockade, and 1 also received corticosteroids. All seven patients who discontinued anabolic steroids had their serum creatinine improved or stabilized and proteinuria reduced. All the patients who had follow-up received ACEi/ARB, and one of them also had prednisone. Only one patient resumed anabolic steroid abuse and suffered relapse of proteinuria and renal insufficiency. In this cohort, clinical features and biopsy findings were more severe compared to traditional post-adaptive FSGS, revealing a possible concurrent direct nephrotoxic effect.

Rifampicin, a common anti-tuberculosis medication, has been blamed quite rarely for minimal-change disease since 1983. Five case reports with biopsy-proven rifampicin-associated MCD were found in the literature from 1983 to 2021 [57,58,59,60,61]. All 5 cases had been on rifampicin for 20 days to 1 month before nephrotic syndrome development and achieved complete remission 4–8 weeks post discontinuation of the drug. Three out of five cases had AKI, and one of them required dialysis, with full renal function recovery post discontinuation, as well. Only 1 patient (the one who required dialysis) was put on steroids with tapering over 3 months. The most commonly described histological picture of rifampicin-related nephrotoxicity so far is tubulointerstitial nephritis. Rapidly progressive glomerulonephritis has also been mentioned [68]. There is not consensus over the pathogenesis of the MCD, probably due to the small number of cases. Whether toxicity is direct or immune-mediated is still unclear [61].

A study of 3 patients having de novo sirolimus and 5 patients having been switched to sirolimus from calcineurin inhibitors post kidney transplantation was conducted in 2007 by Letavernier et al. [52]. Approximately 5 months after sirolimus treatment started, the first 3 patients developed nephrotic-range proteinuria and the other 5 non-nephrotic-range proteinuria. There was no effect on creatinine levels. None of them had primary FSGS, and there was absence of FSGS in renal biopsy performed 3 months before treatment conversion. All patients developed FSGS lesions, but only switched patients exhibited advanced sclerotic lesions. Mean sirolimus trough levels were markedly superior to those currently recommended. All de novo patients and two of the switched group were changed from sirolimus to tacrolimus, leading to a decrease in proteinuria. No patient was switched back to sirolimus. The mammalian-target-of-rapamycin blockage, direct podocyte toxicity and possibly the decrease in VEGF synthesis have been mainly implicated in sirolimus’ podocyte effect.

In 1988, Santella et al. [69] in their case report, proved that lithium does not only cause kidney-concentrating dysfunction and tubulointerstitial nephropathy. They presented 8 cases from the literature with nephrotic-range proteinuria, after being on lithium treatment from 1.5 to 13 months. Renal biopsy showed MCD in five of them, global glomerulosclerosis and tubulointerstitial disease in one and only tubulointerstitial disease in another one. Lithium withdrawal was followed by partial and complete remission and normalization of renal function in the ones who had renal insufficiency. They also described three additional cases diagnosed as FSGS on kidney biopsy. All of them were presented with nephrotic-range proteinuria, with two of them having renal insufficiency as well. The first patient with renal insufficiency who had lithium discontinued achieved partial remission, whereas creatinine remained stable 4 months later (second patient lost on follow-up). The patient with the normal kidney function continued lithium treatment, and he was started on ACEi. Even though he had a slight proteinuria reduction at the beginning, he developed a severe nephrotic syndrome eight months later.

Many years later, Markowitz et al. [51] confirmed the glomerular effect of lithiumby revealing FSGS lesions in combination with tubulointerstitial disease in 12 out of 24 patients with mean duration of lithium therapy of 13.6 years, who were biopsied due to renal insufficiency and proteinuria. The mean follow-up was 30 months. At the time of biopsy, all patients with FSGS lesions had been on lithium already for 13 to 25 years. Secondary FSGS due to hyperfiltration in some cases and direct podocyte toxicity in others are presented as possible explanations for the FSGS picture. The significant degree of foot process effacement suggests a potential direct glomerular toxicity. Despite discontinuation of lithium, 3 (the fourth was already on dialysis the time of biopsy) out of 11 ended up on dialysis 1–5 years post biopsy. Only one patient with high risk of committing suicide, already having been on lithium for 20 years, continued the treatment and ended up on dialysis. The rest of them had decline or stability in creatinine levels post discontinuation. Importantly, these patients had lower initial creatinine levels, due to having had a shorter treatment (12–15 years) compared to the others who ended up on dialysis. Only 1 patient with initial creatinine 1.4 mg/dL (despite 20 years of treatment) had a slight increase 2 years post discontinuation (creatinine 1.7 mg/dL). A serum creatinine of >2.5 mg/dL at the time of biopsy was a significant predictor of progression to ESKD, while the presence of FSGS was not predictive of progression. The study concluded that renal dysfunction due to lithium is very slow, often irreversible despite withdrawal, and early detection is essential to prevent progression to ESKD. 

A broad spectrum of glomerulopathies have been reported secondary to interferon (INF)treatment, such as FSGS, MCD, MN, IgA nephropathy and membranoproliferative glomerulonephritis [70]. Regarding INF-b, FSGS may be a complication of thrombotic microangiopathy itself, possibly via an ischemic mechanism when these two patterns overlap, but generally, the mechanism of each one separately is largely unknown [71]. Possibilities include direct effects of IFN on the podocyte or indirect effects via cytokines, such as IL-6 or IL-13 family members [54,72]. In 2010, Markowitz et al. [54] reported 11 patients who were presented with AKIand nephrotic-range proteinuria after being on treatment with interferonfor various reasons (INF-a, INF-b, INF-c). Specifically, after a median therapyduration of 12.6 months,9 patients had nephrotic syndrome, 2 had non-nephrotic-range proteinuria and 10 had renal insufficiency, with kidney biopsy revealing collapsing FSGS. Follow-up was available for 10 patients, all of whom discontinued interferon. At a mean of 23.6 months, 9 out of 10 patients had improvement in renal function (including 4 who returned to normal-range creatinine) and only 1 ended in ESKD. In terms of proteinuria, one of them achieved complete and two achieved partial remission. Four patients were treated with steroids, including one with partial remission and three who did not meet criteria for remission. Thus, beyond discontinuation of IFN, no additional benefit of immunosuppressive therapy could be detected. 

## 7. Conclusions

In conclusion, there is a variety of medications affecting the glomeruli and podocytes, manifested with various clinical pictures dependent on the affected part. Early recognition of these drug-induced glomerular diseases is mandatory and crucial, because rapid discontinuation of the offending agent is critical to maximize the likelihood of renal function recovery.

## Figures and Tables

**Table 1:** Drug-induced podocytopathies.

First Author	No of Patients	Implicated Medication	Type of Glomerular Disease (Patients)	Clinical Picture (Patients)	Intervention (Patients)	Proteinuria Outcome (Patients)	Renal Function Outcome (Patients)
Hall et al. (1988) [11]	33	Penicillamine	MCD (2)	Proteinuria	Discontinuation	Complete remission	-
Markowitz et al. (2000) [51]	24	Lithium	FSGS (12)	NS (3) NRP (3) NNRP (6) Creatinine rise (12)	Discontinuation(10)	NA	ESRD (4) Creatinine decline (4) Creatinine rise (1)
Letavernier et al. (2007) [52]	8	Sirolimus	FSGS (8)	NRP (3) NNRP (5)	Switch sirolimus to tacrolimus (5)	Proteinuria reduction	-
Perazella et al. (2008) [53]	22	Pamidronate (21)	FSGS (18)	NS (17) NNRP (1)	Discontinuation (19)	Complete remission (4) Proteinuria reduction(5)	ESRD (5) Creatinine decline (7) Creatinine rise (2)
			MCD (3)	NRP (3)			
		Zoledronate (1)	FSGS	NS	Discontinuation		ESRD
Markowitz et al. (2010) [54]	11	INFa-b-c	FSGS (11)	NS (9) NNRP (2) Creatinine rise (10)	Discontinuation (11) Cs (4)	Complete remission (1) Partial remission (2) Proteinuria reduction (7)	Creatinine decline (9) ESRD (1)
Herlitz et al. (2010) [55]	10	Anabolic steroids	FSGS (9)	NS (3) NRP (6) NNRP (1) Creatinine rise (10)	Discontinuation (8) ACEi (7) Cs (1)	Partial remission (4) Complete remission (3)	Creatinine decline (7) ESRD (1)
Izzedine et al. (2014) [43]	29	Sunitinib (11)	Podocytopathy (5)	NRP Creatinine rise (2)	Discontinuation ACEi/ARB	NA	NA
		Axitinib (1)	Podocytopathy				
		Sorafenib (3)	Podocytopathy (2)				
Bakhriansyah et al. (2019) [56]	288	NSAIDS	MCD (15) FSGS (34)	NS	NA	NA	NA
Case Reports (1983–2021) [57,58,59,60,61]	5	Rifampicin	MCD	NS Creatinine rise (3)	Discontinuation	Complete remission	Creatinine decline (3)
Kitchlu et al. (2021) [47]	9	Pembrolizumab(5)	MCD	NS (4) NRP (1) Creatinine rise (5)	Discontinuation Cs	Complete remission (2) Partial remission (1)	Creatinine decline (2) ESRD (1)
		Ipilimumab (2)	MCD	NS (2) Creatinine rise(1)	Discontinuation Cs	Complete remission	Creatinine decline (1)
		Nivolumab (2)	FSGS	NS (1) NNRP (1) Creatinine rise (2)	Discontinuation Cs MMF (1)	Partial remission	Creatinine decline (2)

MCD—minimal-change disease, FSGS—focal segmental glomerulosclerosis, ACEi—angiotensin-converting-enzyme inhibitors, ARB—angiotensin II receptor blockers, NNRP—non-nephrotic range proteinuria, NRP—nephrotic-range proteinuria, NS—nephrotic syndrome, ESRD—end-stage renal disease, NA—not available, MMF—mycophenolate mofetil, Cs—corticosteroids.

## Data Availability

No new data were created or analyzed in this study.
